# A Minimal Turing Test: Reciprocal Sensorimotor Contingencies for Interaction Detection

**DOI:** 10.3389/fnhum.2020.00102

**Published:** 2020-03-24

**Authors:** Pamela Barone, Manuel G. Bedia, Antoni Gomila

**Affiliations:** ^1^Department of Psychology, University of the Balearic Islands, Palma, Spain; ^2^Human Evolution and Cognition Group (EvoCog), University of the Balearic, IFISC, Associated Unit to CSIC, Palma, Spain; ^3^Department of Computer Science and Systems Engineering, University of Zaragoza, Zaragoza, Spain; ^4^Interactive Systems, Adaptivity, Autonomy and Cognition Lab, Aragón Institute of Engineering Research, University of Zaragoza, Zaragoza, Spain

**Keywords:** Turing test, interaction, sensorimotor contingencies, reciprocity, perceptual crossing

## Abstract

In the classical Turing test, participants are challenged to tell whether they are interacting with another human being or with a machine. The way the interaction takes place is not direct, but a distant conversation through computer screen messages. Basic forms of interaction are face-to-face and embodied, context-dependent and based on the detection of reciprocal sensorimotor contingencies. Our idea is that interaction detection requires the integration of proprioceptive and interoceptive patterns with sensorimotor patterns, within quite short time lapses, so that they appear as mutually contingent, as reciprocal. In other words, the experience of interaction takes place when sensorimotor patterns are contingent upon one’s own movements, and vice versa. I react to your movement, you react to mine. When I notice both components, I come to experience an interaction. Therefore, we designed a “minimal” Turing test to investigate how much information is required to detect these reciprocal sensorimotor contingencies. Using a new version of the perceptual crossing paradigm, we tested whether participants resorted to interaction detection to tell apart human from machine agents in repeated encounters with these agents. In two studies, we presented participants with movements of a human agent, either online or offline, and movements of a computerized oscillatory agent in three different blocks. In each block, either auditory or audiovisual feedback was provided along each trial. Analysis of participants’ explicit responses and of the implicit information subsumed in the dynamics of their series will reveal evidence that participants use the reciprocal sensorimotor contingencies within short time windows. For a machine to pass this minimal Turing test, it should be able to generate this sort of reciprocal contingencies.

## Introduction

Alan Turing proposed a famous test in order to study whether machines can exhibit intelligent behavior (Turing, 1950). In the so-called “Turing test,” participants were challenged to tell whether they were interacting with another human being or with a machine. The interaction took place by means of exchanging computer screen messages between the human and the machine, both located in separate rooms. If participants cannot tell apart whether they are communicating with a machine or a human being, Turing reasoned, it must be because the machine exhibits intelligence.

However, the distant verbal conversation of the Turing test is a sophisticated form of interaction, quite different from more basic and typical social exchanges that normally take place among people. These basic forms of interaction are mainly face-to-face and embodied, context-dependent and based on the detection of reciprocal sensorimotor contingencies (Gomila, 2002). In our view, interaction detection in these cases requires the integration of proprioceptive and interoceptive patterns with sensorimotor patterns, within quite short time lapses, so that they are experienced as mutually contingent, as reciprocal. In other words, the experience of interaction takes place when sensorimotor patterns are contingent upon one’s own movements, and vice versa. I react to your movement, you react to mine. When I notice both components, within appropriate time windows, I come to experience an interaction.

Interaction detection makes possible intersubjective experience. Intersubjectivity is the ability to engage in the mutual recognition of mental states without explicitly representing them (Trevarthen, 1979). In second-person interactions, we experience another person’s mind (i.e., agency) in a direct, immediate, non-theoretical, and non-inferential way (Gomila, 2001, 2002; Pérez, 2013; Gomila and Pérez, 2017). This basic understanding is claimed to be the primary form of social cognition in human development (Reddy, 2008; Gomila, 2012); and contrasts with the detached, offline, and inferential way of intentional attributions required to interact in the classical Turing test.

Therefore, we designed a “minimal” Turing task to study how much information is required to detect these reciprocal sensorimotor contingencies and whether we resort to interaction detection –in this basic sense– to tell apart a human from a machine. Our minimal Turing test is inspired in a virtual and simple framework known as the “perceptual crossing” paradigm (Auvray et al., 2009). In the initial perceptual crossing scenario, two participants had to recognize each other in a common unidimensional, virtual space (a line 600 pixels long). They were located in two different rooms, in front of a computer and, while moving in this unidimensional virtual space, they encountered three agents: the avatar of another human, a shadow avatar of the human (also called a “mobile lure,” which repeated the other participant’s movements in another part of the virtual space) or a fixed object. Participants interacted by moving the computer mouse along the line and by receiving tactile stimulation when they crossed over one of the three agents. However, participants could not see the line, their cursor or the avatars that represented each type of agent at any time of the task. They were asked to detect –by clicking– when the tactile stimulation following a crossing had been produced by another human agent.

The main result of the study was that participants did not distinguish between the human and the shadow avatar: the probability of a player clicking the mouse when encountering the person or the mobile lure was not significantly different, although the players’ partners were kept constant (Auvray et al., 2009). However, the correct discrimination between both agents emerged when the authors analyzed the interaction dynamics: As both players were mutually searching for one another, the encounters between the two participants were more frequent than encounters between the participant and the lure, providing an informational cue that might permit a more reliable interaction detection (Auvray and Rohde, 2012).

The perceptual crossing paradigm has been modified in different ways to study the dynamics of human social interactions. Lenay et al. (2011), for example, extended the results from Auvray’s experiment into a richer, two-dimensional scenario, with similar results. On the other hand, Iizuka et al. (2009) expanded the original procedure to test whether pairs of individuals could figure out if an interaction was live or not through a different interface. By moving their fingers left or right in a tactile screen, participants received tactile vibrations when they touched another object in the virtual space. Participants were exposed either to a live interaction with another, always the same, subject or with a recording of a previous live interaction, and were asked to distinguish between them. Although they found it a hard task at the beginning and failed to recognize the two types of interactions, some pairs could develop a turn-taking and signaling strategy that helped them to succeed. However, just 4 out of 10 pairs achieved such a strategy only after tens of trials (Auvray and Rohde, 2012).

In fact, conscious recognition from the other partner only emerged in a version of the perceptual crossing in which participants received different sounds instead of tactile stimulation (Lenay and Stewart, 2012). In this experiment, in which each tone was associated with a type of object, participants could identify the source of the stimulation. The perceptual crossing paradigm has also been employed along the lines of Turing, in robotic and simulation experiments to model the dynamics of interaction detection (see Auvray and Rohde, 2012 for a review). On the other hand, a different strategy has been to develop a visual Turing test, where the interaction is based on joint attention (Pfeiffer et al., 2011).

Recently, an important modification of this framework was implemented by Bedia et al. (2014) who started considering the coupled dynamics of participants’ activities during the test. They devised a program in which a human interacted either with another human agent or with the computer along 10 rounds. The computer could display a shadow avatar of one’s own behavior or an agent with oscillatory movements. Similarly to the study of Auvray et al. (2009), each participant moved the mouse along an invisible line but he/she only heard a sound (instead of receiving tactile stimulation) when he/she crossed over another agent. At the end of each round, participants decided whether they had interacted with a human or with the machine, like in the Turing test.

Bedia et al. (2014) found, as in Auvray’s original study, that the participants were not able of consciously distinguishing between the human avatar and its shadow, and that there was a difference in the pattern of interaction between both conditions. In this case, they found that the probability of having a new stimulation^1^ within 0.5 s after a crossing did differentiate the type of agent involved. In other words, the implicit detection of social contingencies was made evident by a pattern of back and forth movements of both agents around the same point, to generate overcrossings within the half-second time window. This pattern was uncovered by their analysis of the temporal structure of the interaction between two players. A fractal 1/*f* structure (called pink noise) at many timescales of the history of collective interactions emerged only within genuine social interactions (i.e., in the human vs. human case). Moreover, the largest values of the multifractal spectrum width also only appeared in human-human interactions. This distinctive pattern, that exclusively came out in interactions between two human beings, led researchers to argue that genuine social engagement might be better characterized by a structure of cross-scale interactions captured by analyzing fractal 1*/f* scaling and multifractal spectrum (Bedia et al., 2014).

To sum up, previous research with the perceptual crossing paradigm is somehow paradoxical: while it seems to provide a right approach to study interaction detection through social contingencies, it also comes short to prove that our judgments of interaction are based on the reciprocal contingencies detected. In most studies, participants failed to consciously distinguish the shadow agent from the other human participant in spite of evidence of their implicit discrimination in the dynamics of the interaction. The only study that found conscious detection of human interaction through this paradigm (Iizuka et al., 2009) required long series of interaction iteration between fixed pairs of participants, where less than half of participating pairs succeeded. This suggests that the strategies these pairs of participants developed were idiosyncratic, due to the fact that they kept playing with the same partner, rather than resorting to a basic process of reciprocal contingency detection.

In this paper, our main goal is to show that humans do detect interaction through social contingencies. Our second goal is to explore the reasons of the paradoxical results of previous research. In our view, it has to do with the fact that the standard perceptual crossing paradigm includes the three kinds of agents –human, mobile lure, fixed– in each trial, and unimodal feedback only.

As a matter of fact, the motivation to include the shadow agent in the design was meant to parallel the experiment originally devised by Murray and Trevarthen (1985) to study infants’ ability to detect the interaction with an adult. In their pioneer study, they examined the quality of the social interaction between 2 and 3 month-old-infants and their mothers employing a double-video communication system. The baby and the mother were in different rooms and their behaviors were recorded. The infant faced a monitor, which displayed the behavior of his mother, and the mother saw, on her screen, her baby’s behavior. Authors found that babies could distinguish, through their expressive behavior, when they were interacting with their mother from the condition in which they were shown exactly the same sequence but recorded from a previous trial. They reasoned that, in the former condition, babies were responding in real time thus perceiving the mothers’ behavior contingently upon their own, while in the latter, babies lacked the power to influence the images. In the non-contingent scenario, intersubjectivity failed as infants could not engage in the reciprocity of facial expressions with their mothers and, as a result, they showed puzzlement, negative emotional reactions, and reduced eye contact^2^. In the perceptual crossing paradigm, the mobile lure was introduced to include this offline condition: an agent that behaves just like an interacting agent in its global trajectory, but non-contingently upon the behavior of the partner, as its movement depends on that of the human avatar. However, in the perceptual crossing paradigm all conditions are present in each trial, instead of distinguishing online and offline blocks (the only exception is Iizuka et al., 2009).

Therefore, in order to better match Murray and Trevarthen (1985) design what is needed is, first, a condition that closely resembles the offline condition implemented by those authors. This requires an agent with a trajectory identical to one exhibited by an interactive event correctly recognized as such by both participants, but recorded, so that this trajectory is not deployed contingently upon the moves of the participant. If participants decide whether they are interacting with a human on the grounds of the reciprocal sensorimotor contingencies detected, they will judge the offline condition as non-interactive. On the assumption that only human agents can interact in this paradigm, participants will judge that their partner is a human whenever they experience these reciprocal contingencies. And they will judge that their partner is a bot, whenever they do not. For this reason, in our “minimal Turing test” we included trials for each condition, instead of mixing them up.

However, things can be not that simple. For if the participant adopted a passive strategy, one of observing how the other avatar moves, the offline agent could elicit the illusion of interaction, as the pattern of feedback would be identical to a real interaction and participants could experience some reciprocal contingences when interacting with this recorded trajectory and judge that it is a human. Participants should adopt an active strategy in order to perceive the others’ movements as contingent to their own and vice versa. Hence, we tried to provide instructions to participants that fostered this active stance.

On the other hand, Murray and Trevarthen’s (1985) design involved audiovisual contingencies. Previous research with the perceptual crossing paradigm already showed that auditory feedback was more discriminant than tactile stimulation to detect the relevant contingencies. But it may also be the case that the difficulty in consciously distinguishing real interaction from interaction with the mobile lure was because auditory information is not robust enough. Therefore, in our study we also wanted to explore the question of whether the minimal sensory contingencies need to involve more than one modality; in particular, whether audiovisual information is required for robust interaction detection, as in Murray and Trevarthen’s (1985) study. In addition, we also wanted to explore whether participants improve their performance in the auditory feedback block after undergoing the audiovisual block. Thus, we first presented only auditory information about the interaction, then audiovisual stimulation and finally just auditory feedback again, to check whether participants’ performance improved along the task.

In summary, in our minimal Turing test, we modified Bedia et al. (2014) version of the perceptual crossing paradigm to test participants separately in each condition, and through these different feedback blocks. In the first study, participants were exposed either to movements of a human agent, which could be online or offline, or to the movements of a computerized oscillatory agent in three different blocks. In each block, either auditory or audiovisual feedback was provided along each trial, to inform participants when they crossed over another agent. Following previous studies, we first analyzed participants’ recognition about the nature of the other agent by paying attention to their explicit answers. Secondly, we investigated the implicit information subsumed in the interaction dynamics of each participant’s series, like the correlations between the series of two interactors, the time between two crossings, the window of crossings (density of crossings), and the fractal indices, in order to find out whether they tried to solve the task through the crossing patterns they detected. Finally, we asked the participants about their experience with the task. In a second, follow-up study, we simplified the task by eliminating the oscillatory bot, just to focus on the interaction dynamics. We hypothesized that participants are able to detect the social contingencies and to use them to respond to the Turing test question, but wanted to explore which informational conditions turn out to be discriminant enough. Were the information available to the participant insufficient, they would adopt an observational attitude to answer the test question.

## Study 1

### Materials and Methods

#### Participants

A total of 70 participants (55 females) took part in this experiment. Their ages ranged from 20 to 48 years (*M* = 23.15 and *SD* = 4.62). They were recruited from a Psychology class at the University of the Balearic Islands. Participants received credit points for participating in the study. In the lab, they were arranged in groups of six people (11 groups) or in groups of four people (only one group).

#### Experimental Procedure

Each participant entered into a cubicle, wore headphones and sat in front of a computer. As we have six cubicles in the lab, the maximum number of participants that could do the test at the same time was six. Inside the cubicle, they could not see nor hear the other participants. They were instructed on the goal of the study: they had to move their computer mouse in a shared perceptual environment and they were going to hear a sound whenever they crossed over another agent. They had to detect, in each round of the experiment, whether they have interacted with a human or with a computerized agent (for an example of the procedure, see Supplementary Material).

The shared perceptual environment was a virtual, one-dimensional space 800 pixels long (a line) with both ends connected, forming a torus to avoid the singularities induced by the edges, as in Auvray’s original study. Although all movements were available with the computer mouse, only movements from left to right (and vice versa) were considered by the software. When the cursor of a participant crossed the cursor of another agent –either a human or a computerized agent– a collision was perceived because they received an audible stimulus lasting 500 ms. Such audible stimuli were the only environmental perception during some block of trials while the computer screen was black along the whole round. In other block of trials, participants could also see the line and the avatars of each agent.

Specifically, participants started with a training phase in which they could see the line on the screen together with their own avatar (that represented their movements) and the partner’s avatar on it. They performed 4 training rounds and each lasted 15 s. After that, participants received three blocks of experimental rounds. Each block consisted of 9 rounds and each round lasted 30 s. In the first block, participants could not see the line nor the avatars on the screen and just received auditory stimulation when they crossed over another agent. During the second block, participants received audiovisual information in each of the 9 rounds –as in the training phase. Finally, participants received a third block with only auditory feedback (identical to block one).

We decided to provide audiovisual stimulation in the training phase so participants could familiarize with the setup more easily, as they had two sources of environmental stimulation. Block 1 and block 2 were thought to measure how different amount of information would affect such discrimination. Block 3 finally aimed to check whether participants could improve their performance after the second, audiovisual, block.

In each round of the task, participants could encounter one of three possible agents.

1. A “human online agent,” that is, one of the participants from another cubicle. As the task consisted of many trials, the participant was randomly assigned to any other human participant from the group of six to just focus on reciprocal contingency detection as a basic process, and avoid the development of idiosyncratic strategies.
2. A “human offline agent” that consisted in a recording of a previous human-human interaction in which both participants recognized each other as humans. The human offline agent was randomly chosen from an array of series in which two human players previously interacted and correctly recognized each other. Those series were collected from a pilot study and here presented offline.
3. An “oscillatory agent” that exhibited “a sinusoidal behavior (describing a sinusoidal trajectory of 0.5 Hz and 200 pixels of amplitude), predictable and deterministic” (as in Bedia et al., 2014, 4).

As we stated in the instruction, participants were asked to say whether they have interacted with a human or with a computerized agent. In fact, the computer could randomly present either the human offline agent or the oscillatory bot. Therefore, they were unaware of the nature of the computerized agents. The underlying assumptions were that if they experienced an interaction they would respond “person,” that the offline agent would not generate the experience of interaction, and that if the experience of interaction failed to emerge, they would decide from an observational stance.

In the training phase, we only used two types of agents: the human online and another non-reactive bot, which increased its position with a velocity of 30 pixels per second during the whole round. We intentionally employed a different computerized agent that the ones that will appear on the test because we only aimed at introducing the movements of the “machine bots” without showing them the same behavior that were to appear in the task, to avoid any possible habituation or anticipation effect.

In each round of the experiment, participants moved their avatar (by moving their computer mouse) along the unidimensional space, and they heard a sound when they crossed over the other agent or when the other agent crossed over them. At the end of each round, participants had to answer the following question “Who have you interacted with in the last round?” by clicking on one of the answers: “person” or “machine.” Using only auditory information (blocks 1 and 3) or using audiovisual information (training phase and block 2), they had to decide, at the end of each round, whether they interacted with a person or a computerized agent. After they answered, they received feedback about their choice (correct/incorrect). After completing all the procedure, participants were asked about their experience.

#### Coding

On the one hand, we coded participants’ explicit responses at the end of each round as well as the total number of crossings.

##### Participants’ responses

We coded participants’ correct answers at the end of each round, that is, whether they correctly guessed the nature of the other agent. A correct answer was considered when they replied “person” after interacting with the human online agent, and “machine” in the rounds when they interacted with both computerized agents (human offline and oscillatory agent).

##### Number of crossings

We calculated the total number of crossings that each participant executed per round (i.e., the “active crossings,” referring only to those crossings produced by the participant).

On the other hand, we coded different implicit measures in the dynamics of the interaction.

##### Fractal indices

A fractal index was obtained as in Bedia et al. (2014). In a nutshell, we first took the time series of the distance between the two agents in a round. We then computed the agents’ relative velocity (i.e., the first derivative of the distance between the participant and the other agent) to extract whether they are approaching or distancing themselves at each moment of time. Then we used a detrended fluctuation analysis (DFA) algorithm (Peng et al., 2000) to compute the statistical self-affinity in the data series of distance variations (more detail description of the method can be found in the Supplementary Material; also see Bedia et al., 2014). The result is a Beta index (β) that characterizes distinct processes. Values of β close to 0 feature uncorrelated processes (correspond to white noise), values close to 2 exemplify rigid and deterministic processes (brown noise) and values of β close to 1 characterize flexible and adaptable processes (pink noise), that is, processes that are not totally organized but neither totally disorganized.

These background noises refer to intrinsic sources of variability, the intrinsic dynamics of mind and body, and the internal workings of a living being (Van Orden et al., 2003). The interest of this measure is that pink noise has been encountered in biological, physical and cognitive systems and is proposed as a signature of dynamic complexity (Gilden, 2001). These systems are sustained by interaction dominant dynamics; which consist of multiplicative interactions that imply coordination between the different timescales in the system (Van Orden et al., 2005). In the present study, pink noise would only emerge when the participant interacted with the human online, since the mutual detection of sensorimotor contingences would give rise to flexible and adaptable behavior from the partner. Brown noise would emerge when the participant interacted with the oscillatory bot, due to the deterministic movements of this agent; while white noise would better characterize the offline agent since no mutual detection of sensorimotor contingences can emerge along trials.

β was calculated for each series. As a result, we obtained a distribution of “how many β” were for each value within the interval (0, 2) for all the agents.

##### Time between two crossings and window of crossings

We estimated the time it takes to produce another crossing after one took place. This means that, for each crossing in the series, we determine the time until the following one occurs. For example, if there have been crossings at 1000, 3000, 3600, 3800, 3900, 7000, 7300, and 9000 ms of one round, then, the time between crossings would be 2000, 600, 200, 100, 3100, 300, and 1700 ms.

Relatedly, the window of crossing refers to the number of crossings counting after one crossing is produced up to a certain time after this crossing. In a sense, it is the density of crossings (crossings/time; see Supplementary Material for an example of this calculation). It was assumed that the detection of reciprocal contingencies would require rapid back and forth movements around the crossing point by the two partners.

##### Similarity between two series

We calculated the similarity between two series with the crossed-correlation function. This function compares two temporal series and returns a value. The bigger the value, the greater the similarity between both trajectories.

We applied the crossed-correlation function for different time gaps and window spans. A time gap implies comparing two trajectories applying a temporal delay to one of them, to check whether the similarity between both occurred within a specific delay (like an echo). Window spans were chosen based on the crossings. This means that, for each crossing in each round, we took the positions of the two players from their series and calculate their auto-correlation. The correlation of the round is the average of the correlations among all their crossings. Again, the reason of this measure was the expectation that in interactive trials the trajectories of the agents would be similar and the correlation would be higher.

#### Statistical Approach

The design of this experiment involved repeated measures per participant and, in order to account for this characteristic, linear mixed effect models was computed. In order to determine our dependent variable (successful reply), we introduced many predictors: type of agent (human online, human offline, or oscillatory agent); type of block (block 1, block 2, and block 3), participant’s age, gender, β, crossings, and correlation (similarity) between two series.

To avoid a ‘multiple comparisons’ problem, we used bootstrapping tools. In short, bootstrapping methods take data to create new simulated models, providing more robust analyses as well. It has been proved as a generalization of classical multiple comparisons procedures (Berkovits et al., 2000) and its use in some multiple contexts works better than classical methods when it is required a large sample theory and to make the arguments in small samples (Gelman et al., 2012). As noted by Westfall (2011) the bootstrap provides a “simple, elegant generalization of classical multiple comparisons procedures” (p. 1188).

### Results

#### Participants’ Responses and Number of Crossings

Participants greatly succeeded in recognizing the oscillatory bot as a machine in all blocks (binomial two-sided tests, *p*s < 0.001 in the three blocks). Secondly, they recognized the human online as a person in the first two blocks (binomial two-sided tests, block 1: *p* = 0.02, block 2: *p* < 0.001) but not in block 3 (*p* = 0.08). When participants interacted with the human offline agent, they consistently replied that it was a person (binomial two-sided tests, all *p*s < 0.005; see Table 1).

**TABLE 1:** Probability of success and total number of crossings in Study 1.

	BLOCK 1	BLOCK 2	BLOCK 3
**Correct answers**			
Online	0.59	0.70	0.56
Offline	0.32	0.28	0.40
Oscillatory bot	0.66	0.95	0.70
**Number of crossings**			
Online	37.08 (38.7)	27.89 (27.85)	25.71 (24.74)
Offline	32.64 (29.72)	28.38 (26.70)	27.61 (26.22)
Oscillatory bot	32.61 (24.48)	20.80 (27.71)	29.92 (28.19)

*Probability of correct responses about the nature of the other agent and mean (and standard deviation) of crossings in each block and type of agent in Study 1.*

The highest values of recognition appeared in block 2 when participants could also see the avatars on the screen (70 and 95% of successes for online and oscillatory agents, respectively). The comparison of performance in block 3 with block 1 (the blocks providing only auditory information about the crossings) revealed higher recognition of the oscillatory bot (from 66 to 70%) and of the offline agent (from 32 to 40%), while the number of correct answers in the condition of the human online agent barely decreased (from 59 to 56%), but any difference between block 1 and block 3 was significant.

Table 1 also shows the mean number of crossings per block and type of agent. For all type of agents, there were more crossings in the first block than in the other blocks, suggesting an exploratory strategy. In the first block, there were more crossings when the partner was the human online agent than if it was an offline agent or the oscillatory agent. In block 2, crossings decreased in general, but much more when the partner was the oscillatory agent. In block 3, crossings when the partner was human online decreased, and increased for the other two kind of agents. However, crossings did not significantly differ among the types of agent in each block either.

**FIGURE 1:**
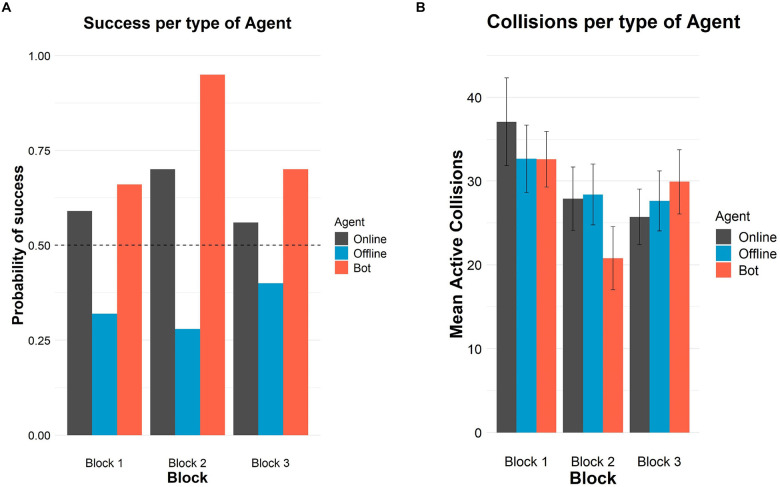
Successes and crossings per type of agent and block in Study 1. **(A)** Probability of success in each block per type of agent. The horizontal dashed line represents chance level (50%). **(B)** Mean number of crossings in each block per type of agent. Error bars depict 95% confidence intervals.

Figure 1 shows the probability of success and crossings.

#### Fractal Indices

**FIGURE 2:**
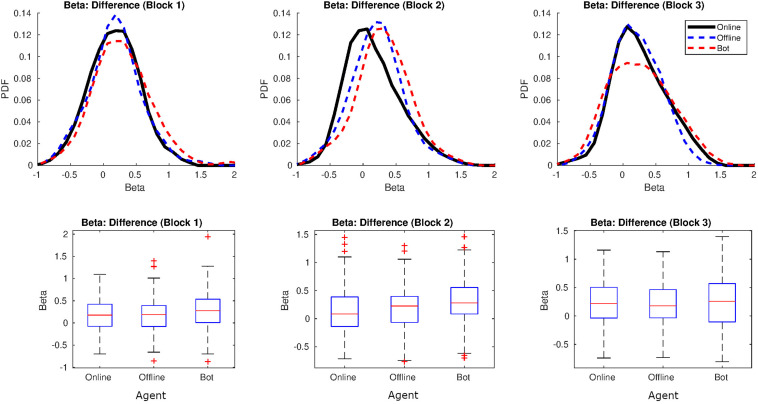
Distribution of β in Study 1. On the top: distributions for the values of β (in black online agent, in blue offline agent, in red the oscillatory bot). On the bottom, representation of the means and variances as boxplots.

Figure 2 shows a graph with the distribution of the values of β per agent and block. Values of β in human-human online rounds were lower in our study, along the three blocks, than the value reported in Bedia et al. (2014; β = 0.86). On the other hand, the β-values in human-human offline series were similar to the β that emerged in the human-shadow condition of Bedia et al. (2014; β = 0.29). In fact, β-values from both human-human online and human-human offline interactions were very similar in our study, and closer to 0 which characterizes uncorrelated processes (white noise) (see Figure 2, at the bottom). For a table with all the values of β per agent and block, see Supplementary Material.

#### Time Between Two Crossings and Window of Crossings

**FIGURE 3:**
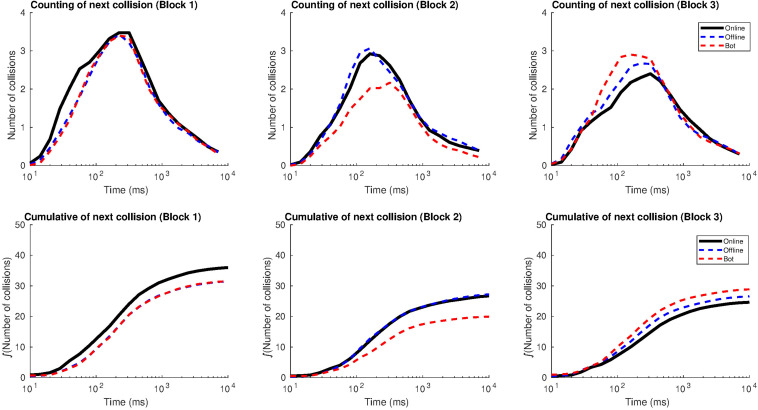
Distribution of the number of crossings according to the window span in Study 1. The distribution has a Gaussian shape. The *x*-axis (time) is shown on a logarithmic scale in order to see the relation between short times (milliseconds) and longer ones (dozens of seconds, which is the length of a round). On the top: number of crossings after one crossing of reference in relation to the time they are produced, for each block and type of agent. On the bottom: number of accumulated crossings as a function of time.

Figure 3 illustrates the distribution of crossings according to the time interval. Graphs on the top show the mean number of crossings, produced after one crossing of reference, in a definite amount of time (considering the previous crossing as a reference). As expected, most crossings took place in between 0.1 and 1 s after the previous crossing (the center of the Gaussian distribution is between 10^2^ and 10^3^ ms).

Graphs at the bottom measure how the crossings were distributed along the round. They represent the accumulated sum of the graphs on top and show that most of the crossings from a round -after one crossing of reference- were distributed in a window span of 1 s (after the initial crossing), generating “gusts of crossings.” In the time interval from 1 to 10 s, the number of accumulated crossings was lower (the sigmoid curve reached a plateau).

The online agent, in Block 1, accumulated more crossings –after one of reference– in the time window of 100 ms than the rest of the agents. The accumulated crossings, after a previous one, sharply decreased in block 3 for the online agent, suggesting a change in how participants moved the cursor.

**FIGURE 4:**
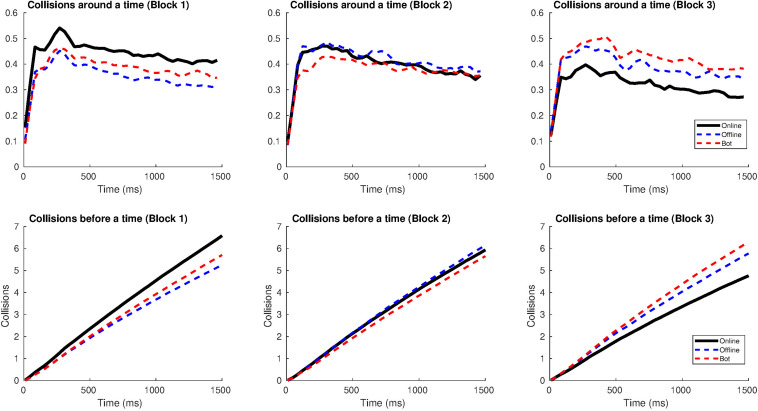
Distribution of the number of crossings as we increase the window span in Study 1. The graphs show how many crossings are produced in the window span from one crossing until a specific amount of time.

Figure 4 reports the distribution of crossings as we increase the window span, between 500 and 1500 ms. In general, the number of crossings diminished for the online agent from block 1 to block 3 (in line with the findings from the previous graph). In block 1, when a participant crossed over the online agent, the following crossings increased up to seven within the 1500 ms window span while, in block 3, they added up to four more crossings. The opposite pattern followed the distribution of crossings for the offline and oscillatory agents, since the crossings after the first one occurred slightly increased from block 1 to block 3 (from five to six crossings in the 1500 ms window span).

#### Similarity Between Two Series

**FIGURE 5:**
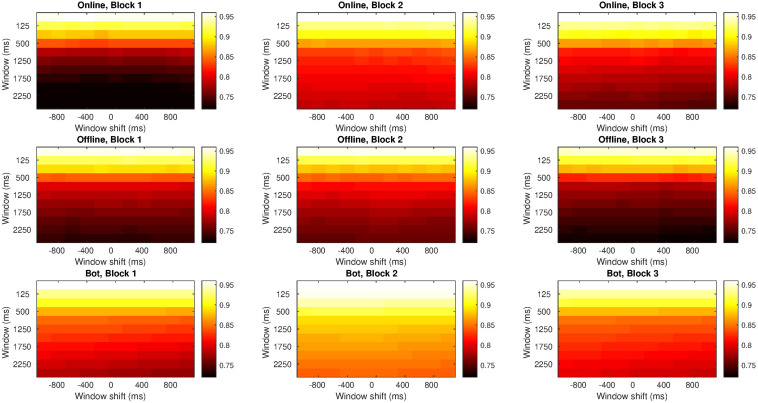
Correlation indices for different windows span and delay times in Study 1. Each row refers to a type of agent (human online, human offline and oscillatory bot) and each column refers to either block 1, block 2, or block 3. Inside each graph, the horizontal axis is the delay applied (from –1 s to 1 s), with the central column showing no delay at all. Vertical axis alludes to the window span, from 50 ms (at the top) to 2500 ms (at the bottom). Light colors indicate higher correlation and dark colors indicate lower correlation. All the graphs show the same scale of colors; then, colors can be compared among graphs.

Figure 5 shows the similarity between participants’ series. On the one hand, light colors appeared at the top of the diagrams indicating a high correlation in small window spans. The smaller the windows span, the shorter the series were, and more similar to each other. On the other hand, colors got darker from the top to the bottom, which means that similarity gradually decreased as the window span increased.

Comparing the three blocks, the greatest similarity occurred in block 2 when the avatars were visible. But when considering the type of agent, regardless of the block, there was a greater similarity between the participant’s and the bot’s series than with the other agents.

In bigger window spans, the similarity with the human online series decreased: we can see that the colors at the bottom in block 1 are darker (i.e., less similarity) than the colors at the bottom in block 3. In big windows span, however, the similarity with the human offline series decreased along the study: the colors at the bottom of block 1 are lighter (i.e., more similarity) than the colors at the bottom of block 3.

Since there was no difference across the distinct delays applied (colors are uniformly distributed for diverse time gaps), we can employ the values with no delay. It means that we took the correlation index for window span with no delay applied to the trajectories in the statistical model we will present in section “Statistical Analysis” (for a table with all the correlation indices see the Supplementary Material).

**FIGURE 6:**
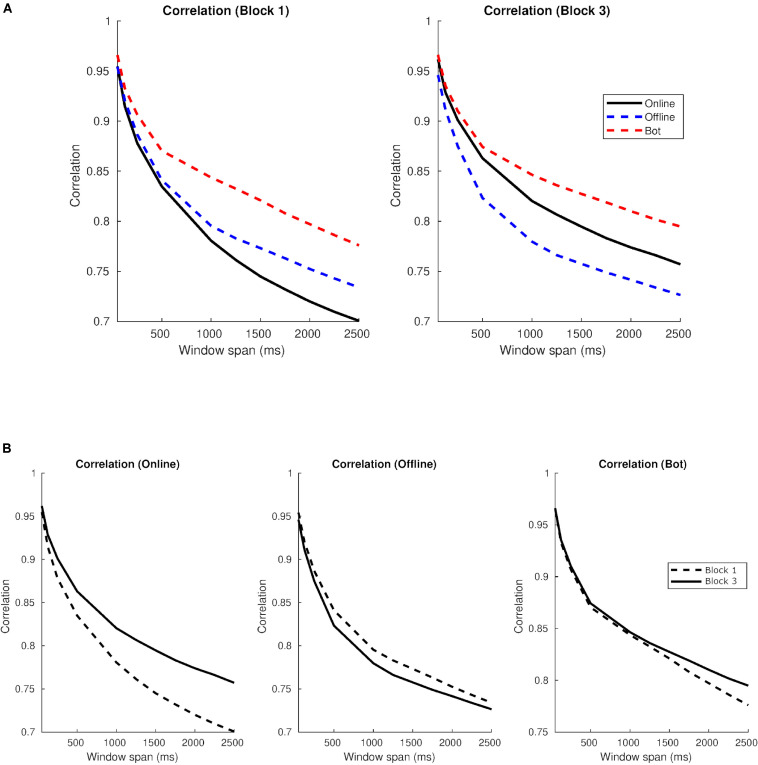
Correlations in auditory blocks and per type of agent in Study 1. **(A)** Correlation indices in block 1 (left) and block 3 (right) per type of agent as a function of the window span. **(B)** Correlation indices per online agent (left), offline agent (medium) and oscillatory bot (right) in block 1 and block 3.

Figure 6 takes the correlation values between two trajectories with no delay applied and shows how these indices change when different window spans are considered. On the one hand, Figure 6A shows that the highest correlations were found with the bot both in blocks 1 and 3. While in block 1 the correlation with the online agent was inferior than the correlation with the offline agent, in block 3 correlations with online were higher than the offline. On the other hand, Figure 6B indicates that the correlation values were higher in block 3 than in block 1 for the human online in all temporal scales. A similar pattern emerged with the oscillatory bot, with clearly higher correlations in block 3 in windows spans bigger than 1.5 s. The offline agent presented the opposite pattern since correlations decreased in block 3 in comparison to block 1.

#### Statistical Analysis

We first analyzed all the rounds. Many of the predictors of our model (age, gender, β and density of crossings) did not discriminate when the participant gave the correct answer (all *p*-Values > 0.05). Correlation indices between series with windows at 500 and 1200 ms seemed to discriminate participants’ correct answer [Corr500, *t*(1815) = −2.14, *p* = 0.03; Corr1200, *t*(1815) = 1.8, *p* = 0.06]. However, the *p*-Value for the correlations for the second window span was not significant.

Secondly, we analyzed the results according to the type of agent and each block. Results, however, did not show any significant variable that predicts the correct response (all *p*s > 0.05) (see Supplementary Material for the detailed results).

#### Debriefing

After the procedure was over, participants answered three questions:

– Describe briefly how you played the game.
– How have you decided that the agent was a human?
– How have you decided that the agent was a machine?

We grouped the responses into three categories:

(1) Reciprocity-based decisions: the participant explicitly said to ground her response on the reciprocity of movements, and/or the contingency between her movements and the other player’s movements to decide her reply. They said, for example, that they searched the partner and then waited to see whether it reciprocated.
(2) Partially reciprocal decisions: the participant said to take reciprocity into account sometimes, but not always, and/or not in a consistent way. For instance, one said that she judged the agent was human because it responded to the participant’s actions and because it moved randomly.
(3) Non-reciprocal responses: the participant said her decision was based on other features related to the pattern of sounds, the total number of crossings during a trial or the velocity of the movements.

In this study, only 11% of the participants’ responses corresponded to category 1, 16% to category 2, and 73% to category 3.

### Discussion

In our first study, participants explicitly differentiated the interaction with the human agents –online and offline– from the interaction with the oscillatory agent. Although they correctly identified the oscillatory bot as a machine, they treated the online and the offline agents as two instances of the same type of agent. When they interacted with either of them, they explicitly answered that they had interacted with a person. In a way, they were right, as the offline sequence was the replay of the previous online movements of a person. This suggests that responses were based on an observational stance rather than on the detection of the social contingencies available. Thus, the decisions took into account preferentially the degree of complexity of the sequence, responding “machine” when it exhibited a regular and predictable patters, and “person” when it did not. This hypothesis is reinforced by the fact that they performed alike across the three blocks. The audiovisual feedback of block 2 made no difference.

Interestingly, the different measures of an implicit interaction detection did not show that participants moved differently in the online vs. offline human conditions. Neither the features of the trajectories, the amount of crossings, nor the fractal analysis revealed a difference between online vs. offline agents across the blocks. Although the β for the interaction with the offline agent is similar to the index found in Bedia (a value that features uncorrelated processes), comparable indices were obtained for the other agents, again suggesting that our participants were trying to tell the nature of the other agent on the grounds of its global temporal pattern of sounds rather than on its contingency upon the participant moves.

On the other hand, the similarity between the two trajectories was greater in block 2 than in the other blocks, suggesting that the visual information available increased the matching of movements to those observed, but not enough to influence participants’ response for that block. We found a greater correlation between the trajectories of agents at the 500 ms window span, suggesting that it is around this half second that contingencies may generate the experience of interaction, even if the participants’ responses were not based on such experience, but on a periodical trajectory of the other agent.

In summary, participants’ explicit responses in our first study were not based on the perceived contingency of the interaction, but presumably on the pattern of the other agent’s movements (“if it is predictable and periodic, it is a machine; if not, it is a human”). As a matter of fact, the evidence indicates that they were not even able to implicitly discriminate between the online and offline conditions –in contraposition to previous results with the perceptual crossing paradigm. The reason for this, we submit, is that the offline condition can generate the experience of interaction, as the participant may respond contingently on the crossings detected. In addition, the fact that we provided two response options, “person” or “machine,” pragmatically suggested to our participants that there were just two kinds of agents, inducing them to adopt an observational strategy over an interactive one; while, in fact, we presented three different kinds of moving agents in the set up. Similarly, the feedback provided after each trial, with so many errors, may have fostered the adoption of the “periodic pattern = machine, non-periodic = human” heuristic. The only possible way for participants to judge the offline cases as “machine” ones was by noticing their lack of responsivity to the participant moves, but either they did not look at this kind of information at all or the information available was not enough to detect such contingencies.

## Study 2

In order to explore this *post hoc* hypothesis, we designed a second study. To better address the participants’ attention to the pattern of interaction, we introduced modifications to the experimental paradigm. First, we only employed two types of agents: the human online and the human offline. In this way, given that the two kind of trajectories the participant could met were equivalent in complexity and unpredictability from an observer point of view, the only way to respond correctly would be by looking for reciprocal contingencies: moving and checking whether the other agent’s movement was reactive to one’s own movement. In particular, whether the pattern of back and forth movements of both agents around the same point emerged, and whether the participants could use this interaction pattern to respond to this new version of the “Turing test.”

Secondly, we modified the way of giving the interrogation at the end of each round. Instead of choosing between two responses (person or machine), participants will decide the nature of the agent they have just interacted by selecting a point on a Likert scale. In this way, confidence in the response could be measured. No feedback about their answer was provided to prevent the development of strategies through trial and error along the procedure.

### Materials and Methods

#### Participants

We tested 50 participants, recruited from a Psychology class, at the University of the Balearic Islands. Participants received credit points for participating in the study. In the lab, they were arranged in groups of 4 people (12 groups) and only one group of 2^3^. Two participants were excluded from the analysis because they had already participated in the first experiment. Our sample, then, consisted of 48 participants (9 males). Their ages ranged from 20 to 40 years (*M* = 23.45 and *SD* = 4.93).

#### Experimental Procedure

The procedure was similar to Study 1. After each participant entered into the cubicle and wore the headphones, 4 training trials were administered. During the training phase, the participant could see his/her avatar and his/her partner’s avatar on a line on the screen. Each trial lasted for 15 s. At the end of each round, they had to detect whether they interacted with a human online or with a human offline. They replied by selecting one point on a 7-point Likert scale which represented how sure they were about the nature of the other agent. For instance, if they were completely sure that they had interacted with the human online, they chose one extreme of the scale that represented the response “I am completely sure it was online.” If they were completely sure that they had interacted with the human offline, then they chose the other extreme of the scale that depicted the opposite reply; that is, “I am completely sure it was offline.” In case they did not know the nature of the other agent, they chose the middle of the scale. Participants could also choose other two possibilities between the middle and each extreme of the scale. They did not receive feedback after each trial.

Each participant was tested along 6 rounds in block 1 (in which auditory feedback was provided), 6 rounds in block 2 (with audiovisual feedback) and 6 rounds in block 3 (again, only supplying auditory feedback). In each block, participants interacted with either a human online or a human offline agent in rounds that lasted 30 s. Like in the training phase, they had to decide, at the end of each round, whether they interacted with a human online or offline.

#### Coding

Like in Study 1, we coded participants’ responses, the total number of crossings, fractal indices, the time between two crossings, the window of crossings, as well as the similarity between each pair of trajectories (the participant and the corresponding counterpart). In this case, participants’ responses were considered as correct ones when they replied “online” after interacting with the human online agent, and “offline” in the rounds when they interacted with the human offline agent.

#### Statistical Approach

We again computed linear mixed effect models with the following predictors: type of agent (human online or human offline); type of block (block 1, block 2 and block 3), participant’s age, gender, β, crossings, and correlation (similarity) between each pair of trajectories.

### Results

#### Participants’ Responses and Number of Crossings

In block 2, participants correctly identified the online agent (binomial two-sided test, *p* < 0.001). For the offline agent, although the probability of success was 0.53, it was not different from chance level (binomial two-sided test, *p* = 0.5). In all other blocks, participants’ probability of success was not different from chance level for any type of agent (binomial two-sided tests, all *p*s > 0.56) and there was no difference in performance between block 1 and block 3 (see Table 2).

**TABLE 2:** Probability of success and total number of crossings in Study 2.

	BLOCK 1	BLOCK 2	BLOCK 3
**Correct answers**			
Online	0.5	0.73	0.47
Offline	0.49	0.53	0.49
**Number of crossings**			
Online	42.92 (25.85)	25.65 (21.25)	36.99 (27.10)
Offline	38.88 (29.12)	27.59 (25.17)	30.57 (22.70)

*Probability of correct responses about the nature of the other agent and mean (and standard deviation) of crossings in each block and type of agent in Study 2.*

Considering only the online agent, the probability of success increased in block 2 (when participants could see the avatars on the screen) compared to block 1 and it decreased again in block 3. For the offline agent, the recognition rate was at chance level (between 0.49 and 0.53) in all blocks.

**FIGURE 7:**
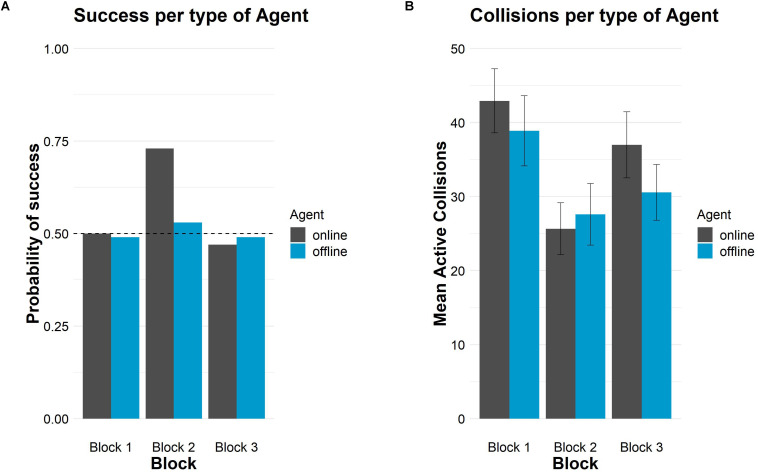
Successes and crossings per type of agent and block in Study 2. **(A)** Probability of success in each block per type of agent. The horizontal dashed line represents chance level (50%). **(B)** Mean number of crossings in each block per type of agent. Error bars depict 95% confidence intervals.

On the other hand, Table 2 also shows the mean number of crossings that participants performed per block. In general, participants produced more crossings in block 1, for both types of agents, than in the rest of the blocks and they caused the fewest crossings in block 2. Participants collided more with the online than with the offline agent in both auditory blocks. In block 2, however, the mean number of crossings was similar for both agents. The probability of success and crossings are shown in Figure 7.

#### Fractal Indices

**FIGURE 8:**
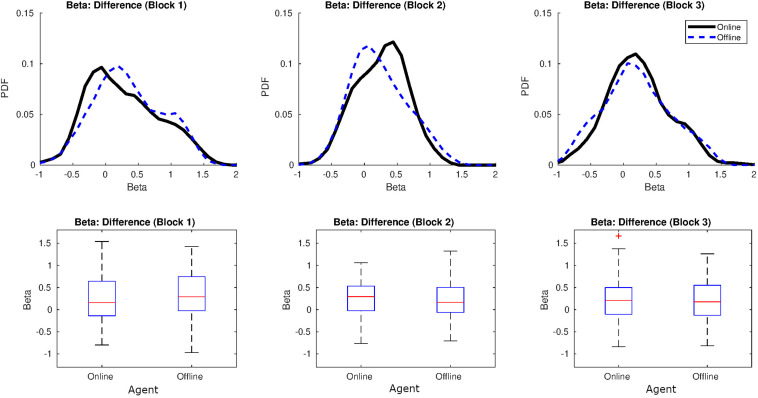
Distribution of β in Study 2. At the top: distributions for the values of β (in black online agent, in blue offline agent). At the bottom, representation of the means and variances as boxplots.

Figure 8 shows a graph with the distribution of the values of β per agent and block. Values of β for human-human online rounds were again lower than the values reported in Bedia et al. (2014; β = 0.86), while the values of β for the human offline agent were similar to the values of the shadow in Bedia et al. (2014; β = 0.29). Once more, β were practically identical in online and offline rounds and closer to 0 which characterizes uncorrelated processes (white noise; see Figure 8, bottom). For a table with all the values of β per agent and block, see Supplementary Material.

#### Time Between Two Crossings and Window of Crossings

**FIGURE 9:**
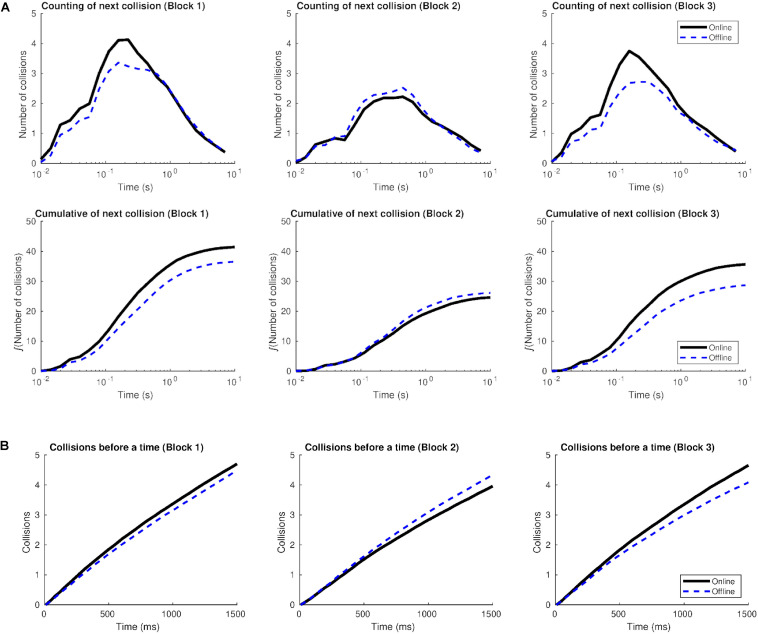
Distribution of the number of crossings according to the window span in Study 2. **(A)** The distribution has a Gaussian shape. The *x*-axis (time) is shown on a logarithmic scale in order to see the relation between short times (milliseconds) and longer ones (dozens of seconds, which is the length of a round). At the top: number of crossings after one crossing of reference in relation to the time they are produced, for each block and type of agent. At the bottom: the number of accumulated crossings as a function of time. **(B)** Distribution of the number of crossings as we increase the window span. The graphs show how many crossings are produced in the window span from one crossing until a specific amount of time.

Similar to Study 1, most crossings took place between 0.1 and 1 s after the previous crossing, as the center of the Gaussian distribution is between 10^2^ and 10^3^ ms (see Figure 9A, top). Also, most of the crossings from a round –after one crossing of reference– were distributed in a window span of 1 s (after the initial crossing), generating “gusts of crossings” (Figure 9A, bottom). In the time interval from 1 to 10 s, the number of accumulated crossings was lower (the sigmoid curve reached a plateau).

In blocks 1 and 3, more crossings occurred in the online agent condition than in the offline one, for the time interval lesser than 1 s. In block 2, the accumulated crossings –after one of reference– sharply decreased for both kinds of agents, in comparison to block 1, suggesting a greater role for the visual information.

Figure 9B shows the distribution of crossings as we increase the window span, between 500 and 1500 ms. In general, the number of crossings remained similar for the online agent from block 1 to block 3. In both blocks, when a participant crossed over the online agent, the following crossings summed up to four within the 1500 ms window span. This pattern was very similar for the offline agent in block 1 while it was slightly inferior in block 3: the offline reached at about 3 crossings, after one occurred, during the 1500 ms window span.

#### Similarity Between Two Series

**FIGURE 10:**
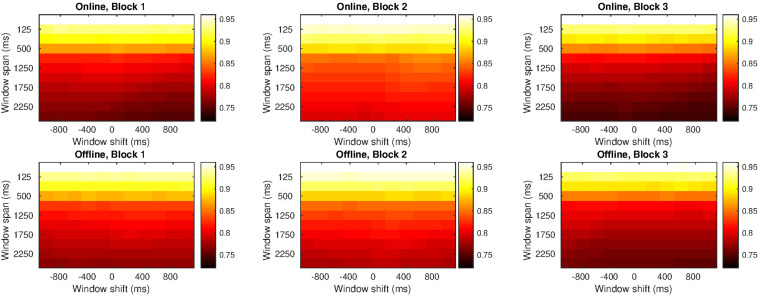
Correlation indices for different windows span and delay times in Study 2. Each row refers to a type of agent (human online and human offline) and each column refers to either block 1, block 2, or block 3. Inside each graph, the horizontal axis is the delay applied (from –1 s to 1 s), with the central column showing no delay at all. Vertical axis alludes to the window span and it ranges from 50 ms **(at the top)** to 2500 ms **(at the bottom)**. Light colors indicate higher correlation and dark colors indicate lower correlation. All the graphs show the same scale of colors; then, colors can be compared among graphs.

Like in Study 1, correlations between series were very high when considering small time windows (in other words, the series of both interacting agents maximally resemble each other). This similarity slowly decreased as bigger time window were considered (see Figure 10). Also, like in Study 1, the greatest similarity occurred in block 2 when the other agent’s movements were visible to the participant.

Colors were similarly distributed in blocks 1 and 3, indicating that correlation between trajectories were similar in both blocks, regardless of the type of agent involved. This suggests that, after the increase in correlation that occurred in the second block, the indices returned to the levels of block 1.

Since there was no difference across the distinct delays applied (colors were again uniformly distributed for diverse time delays), we employed the values with no delay. It means that we took the correlation index for the window span with no delay applied to the trajectories in the statistical model we will present in section “Statistical Analysis.”

**FIGURE 11:**
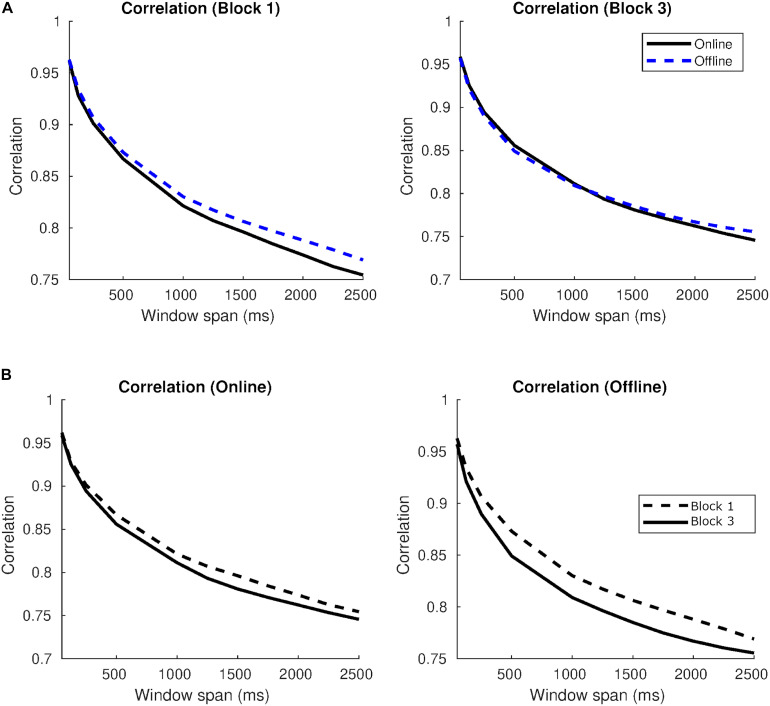
Correlations in auditory blocks and per type of agent in Study 2. **(A)** Correlation indices in block 1 (left) and block 3 (right) per type of agent as a function of the window span. **(B)** Correlation indices per online agent (left) and offline agent (right) in block 1 and block 3.

Figure 11 also takes the correlation values between two trajectories with no delay and represents how these indices change when different window spans are considered. On the one hand, Figure 11A shows that, while in block 1 the correlation with the online agent was slightly lower than the correlation with the offline agent, in block 3 the correlation indices were similar for both types of agents. On the other hand, Figure 11B indicates that correlations were higher in block 1 than in block 3 for both online and offline agents in all window spans.

#### Statistical Analysis

We built a model containing several predictors. We found, on the one hand, that block 2 discriminated participants’ correct answer [*t*(837) = 3.53, *p* < 0.001]. On the other hand, all the other predictors (type of agent, age, gender, beta, density of crossings and correlation indices) did not discriminate when the participant gave the correct answer (*p*s > 0.05; see Supplementary Material for the detailed results).

These results differ from what we found in Study 1: now the audiovisual information provided in block 2 allowed participants to correctly distinguish both kinds of agents.

#### Debriefing

As in Study 1, at the end of the procedure we asked the same three questions about their experience in the test to the participants:

– Describe briefly how you played the game.
– How have you decided that the agent was online?
– How have you decided that the agent was offline?

This time, 65% of participants’ responses corresponded to category 1 (“reciprocity-based” responses), 25% to category 2 (“partially reciprocity-based” responses), and 10% to category 3 (“non-reciprocity based” responses). We take this as evidence that our second study managed to induce participants to rely on interaction detection, even if the information available was not discriminant enough, except for the audiovisual block.

#### Confidence in the Response

We also analyzed the performance in the rounds that participants reported to feel completely sure of their response. We took into account the extreme points of the 7-point Likert scale that represented the responses “I am completely sure that I interacted with the Online agent” (1) and “I am completely sure that I interacted with the Offline agent” (7) and selected the rounds in which participants gave that reply.

A total of 303 series, out of 860, qualified as maximally confident ones. Most participants (45 out of 48) were represented in this subset. In those trials, the participants correctly identified the online agent (binomial two-sided test, *p* < 0.001) and the offline agent (binomial two-sided test, *p* = 0.05) in block 2. Their probability of success was not different from chance level for any type of agent in blocks 1 and 3 (binomial two-sided tests, all *p*s > 0.23). In general, the performance was different from chance level only in block 2, although it was always over 0.5 in all blocks (see Table 3).

**TABLE 3:** Probability of success and total number of crossings.

	BLOCK 1	BLOCK 2	BLOCK 3
**Correct answers**			
Online	0.6	0.8	0.59
Offline	0.51	0.63	0.53
**Number of crossings**			
Online	47.28 (27.64)	27.38 (13.99)	39.78 (30.28)
Offline	45.86 (51.14)	27.59 (29.58)	30.10 (25.60)

*Probability of correct responses about the nature of the other agent and mean (and standard deviation) of crossings in each block and type of agent.*

**FIGURE 12:**
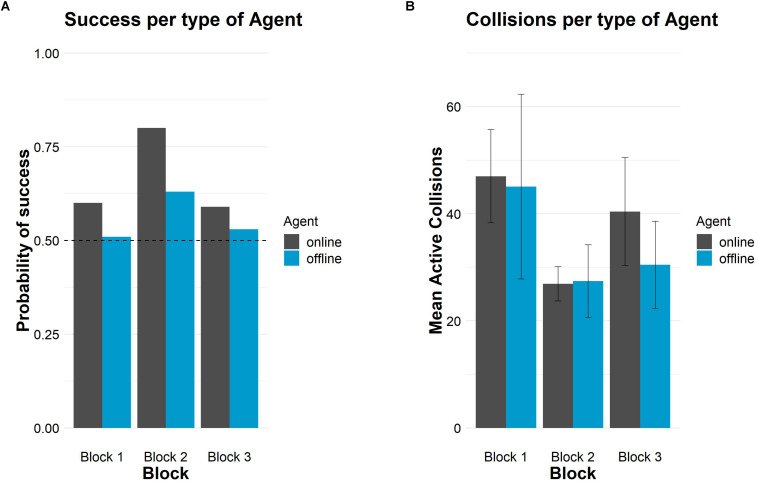
Successes and crossings per type of agent and block for the self-confident sample. **(A)** Probability of success in each block per type of agent. The horizontal dashed line represents chance level (50%). **(B)** Mean number of crossings in each block per type of agent. Error bars depict 95% confidence intervals.

Regarding crossings, self-confident participants produced more crossings in block 1, in the two conditions, than in the rest of the blocks. As in Study 1, they produced, on average, fewer crossings in block 2 than in the rest of the blocks (see also Table 3). In general, there were more crossings with the online than the offline agent in blocks 1 and 3. In block 2 there were the same number of crossings for both types of agents. Figure 12 shows the probability of success and the crossings for online and offline agents in the three blocks.

The type of block had a significant effect on crossings, χ^2^(7) = 16.39, *p* < 0.001, but not the type of agent, χ^2^(8) = 0.50, *p* = 0.47. Most important, the block × agent interaction was not significant, χ^2^(10) = 1.46, *p* = 0.48.

### Discussion

The changes to our experimental paradigm introduced in our second study were able to reveal the use of reciprocal contingencies to detect an interaction and to explicitly respond the question task, but only when the available information was sufficient to do so: participants were able to correctly distinguish whether they were interacting with another person when they could see the other moving as they moved, but not when just hearing a sound when a crossing took place. To put it another way, participants responded at chance level when they could not tell apart whether an interaction was taking place or not, as it happened in the auditory condition. The perceptual crossing paradigm, which only provides acoustic (or tactile) feedback of the crossing, does not seem to provide enough discriminant information to detect social contingencies.

This insufficiency is made clearer when it is realized that the participant can adopt either an active or a passive strategy in addressing the task. He/she may move along the axis trying to cross over the other agent, to see what happens next (i.e., active strategy), or he/she may wait for a crossing over to happen, to see whether the other agent moved back, generating the characteristic series of crossings around a point (i.e., passive strategy). Moreover, they may combine both strategies in a single trial. But given that the offline agent in the second study was a trajectory that had already been correctly deemed as human, they might have generated an illusion of interaction, as these contingencies were in fact available in the participants –except in the audiovisual condition, where the participants correctly classified the agent, on the unique evidence available, the interactive one. In this way, Study 2 also confirms our hypothesis that interaction detection involves the detection of contingencies within a short window span, inferior to 1 s –basically around 500 ms, as found in Study 1.

## General Discussion

In this article, we have shown that individuals can resort to social contingencies to respond in a minimal Turing test. Based on a new version of the perceptual crossing paradigm, we also investigated in two studies how much information is required to reliably detect the reciprocal sensorimotor contingencies in social interactions and, therefore, recognize the nature of the other agent.

In particular, we presented participants with movements of a human agent, either online or offline, and movements of an oscillatory agent in three different blocks. In each block, participants received either auditory or audiovisual feedback along each trial. Analysis of participants’ explicit responses and of the implicit information subsumed in the dynamics of their series revealed that participants use the reciprocal sensorimotor contingencies of the interaction in very specific scenarios.

The results of Study 1 showed that although participants differentiated the human agents from the oscillatory agent and correctly identified the latter one as a machine, they judged both the online and the offline agents as persons, and their performance did not improve in the audiovisual condition. Analysis of the implicit measures during the interaction revealed no specific pattern for each type of agent. According to these results, participants may have used the periodicity of the other’s movements to base their response instead of detecting the interaction. The implicit measures of interaction agree with the participant’s reports at the end of the procedure.

The instructions given in Study 1 failed to induce the participants to look for the cues of interaction: while they expected to interact either with a human or a machine, we presented them with three different traces that corresponded to three types of agents (online, offline, and the oscillatory bot). In order to better induce the participant to pay attention to the interaction cues, in Study 2 we eliminated the oscillatory bot and changed the question to tell whether the other agents were online or offline. We also adjusted the way participants replied at the end of each round: they had to select, on a 7-point Likert scale, how sure they were if the other agent was online or offline. The results showed that participants correctly identified the online agent in the audiovisual condition but they failed to recognize the offline agent in all blocks, even when they could see the avatars on the screen.

Only when we analyze the performance of the participants who were completely sure of their reply, correct recognition of both the online and the offline agents in the audiovisual condition emerged. Therefore, our results show that participants can base their responses on the coupled dynamics of interaction. However, auditory signaling the crossing is not enough to tell apart real interaction from a previously recorded, rightly recognized as interactive, trajectories. This is congruent with previous studies in the perceptual crossing framework. They relied on one modality only (tactile stimulation or auditory feedback) and kept that stimulation constant along the study and found the difficulty in discriminating the avatar of the human agent from the mobile lure. Associating different tones to each type of agent may be more informative (Lenay and Stewart, 2012) but might also induce participants to pay attention to the sounds themselves instead to the pattern of the interaction.

Therefore, our study shows that participants are able to use reciprocal sensorimotor contingencies of the interaction, even if the minimal information available may require more than one sensory modality. As a matter of fact, only when both auditory and visual cues are provided participants became reliable in distinguishing the type of agent they are interacting with. This finding is congruent with the intersensory redundancy hypothesis (Bahrick and Lickliter, 2012) according to which amodal properties are best perceived when simultaneous co-occurrence of stimulation across different sense modalities takes place. In our task, the temporal synchrony of the interaction was detected in the audiovisual scenario when participants simultaneously perceived visual and auditory information of the interaction. Such intersensory facilitation happens because “redundantly specified, amodal properties are highly salient and [thus] detected more easily in bimodal synchronous stimulation than […] the same amodal properties in unimodal stimulation” (Bahrick and Lickliter, 2012, 188).

Interestingly, we did not find a significant difference between the first and the third block in any of the studies. The audiovisual block 2 did not have an effect in the last block, again suggesting the insufficiency of unimodal information for interaction detection.

Our studies also confirmed the utility of the implicit measures of interaction introduced by Bedia et al. (2014). The correlation between the series of two players, showed that the series resembled more to each other in the audiovisual condition at the 500 ms window span. This means that the participants tended to assimilate their trajectories to those of the agents they were interacting with when they could see them. It is also around this half-second that contingencies may generate the experience of interaction. Interestingly, developmental studies revealed that social contingency between different response modalities of infants and their mothers can also be appreciated in a similar window span equal to or less than 1 s long (Dominguez et al., 2016; Español et al., under revision). On the one hand, newborns’ and maternal vocalizations occurred within a 1-s window (Dominguez et al., 2016) and, on the other hand, maternal responsiveness through patterns of imitation and affect attunement to the infant’s signals during the first 10 months also unfold during these short time windows (Español et al., under revision). In general, the window span for cycles of reciprocity using different response patterns does not seem to vary through development.

However, fractal analyses did not result in any specific pattern per type of agent. This null result may be partially explained by the audiovisual condition. In the original study of Bedia et al. (2014) “the emergence of a 1*/f* structure for genuine social interaction is something that happens only in the shared space between the two subjects, and the process cannot be reduced to the individual dynamics of any of them” (p. 11). In our study, the shared space was not maintained exactly constant along the whole experiment since the feedback was different in each block. This difference seems to be critical for this measure.

Finally, the introduction of the human offline agent may have caused some difficulties in the procedure. If we compare it with the previous shadow and lure bots from other perceptual crossing experiments, the behavior of our offline agent was more complex: it was able to create the illusion of interaction because it was originally the outcome of a previous contingent interaction between two humans. This additional complexity may have confused the participants, making it very difficult to discriminate between the online and the offline cases and struggling also the way they interacted with each agent. Although previous research showed that the detection of the offline agent is possible in a tactile setup with pair of participants (Iizuka et al., 2009), this achievement required tens of trials. We did not run the study along such many trials to test whether recognition finally emerged over extended periods of interactions, but we think that it possibly does.

There is an additional limitation in our study: although a participant interacted with one agent at a time, the behavior of the human online agent did not correspond to the *same* human participant along the trials. As we performed a group experiment (groups of 4 or 6 participants in a row), each trial in which a participant interacted with a human online was randomly assigned to a group, so he/she never interacted with the same counterpart in the online condition. That is, each participant encountered up to 5 different people in Study 1, or up to 3 human counterparts in Study 2. Previous experiments studied how the detection of agency emerged in pair of participants as the other human of this pair was the same person during the whole setup (except for Bedia et al., 2014). As each person can display different strategies and behaviors, this source of variation could increase the difficulty of the task. More research is needed to delve into each of these issues.

## Data Availability Statement

The raw data supporting the conclusions of this article will be made available by the authors, without undue reservation, to any researcher.

## Ethics Statement

This study was carried out in accordance with the recommendations of ethical guidelines of the Research Ethics Committee of the University of the Balearic Islands. All participants gave written informed consent in accordance with the Declaration of Helsinki. The protocol was approved by the Research Ethics Committee of the University of the Balearic Islands.

## Author Contributions

PB and AG conceived of the presented idea, designed the study, and wrote the manuscript. MB worked out almost all of the technical details of the analyses. PB carried out the experiments. PB and MB performed the analyses and designed the figures and tables. AG supervised the project.

## Conflict of Interest

The authors declare that the research was conducted in the absence of any commercial or financial relationships that could be construed as a potential conflict of interest.

## Footnotes

1 That is, the percentage of crossings that are followed by a second crossing.

2 Other studies have also acknowledged that babies seem to use social contingency to recognize an interactive partner (Bigelow et al., 1996; Nadel et al., 1999; Bigelow and Decoste, 2003; Stormark and Braarud, 2004; Field et al., 2005; Okanda and Itakura, 2005)

3 Although our lab counts with 6 cubicles, one computer was broken. Then, we arranged the experiment in groups of 4 people considering that the setup is programed to work with an even number of participants.
