# Corrigendum: A minimal Turing test: reciprocal sensorimotor contingencies for interaction detection

**DOI:** 10.3389/fnhum.2024.1507296

**Published:** 2024-10-17

**Authors:** Pamela Barone, Manuel G. Bedia, Antoni Gomila

**Affiliations:** ^1^Department of Psychology, University of the Balearic Islands, Palma, Spain; ^2^Human Evolution and Cognition Group (EvoCog), University of the Balearic, IFISC, Associated Unit to CSIC, Palma, Spain; ^3^Department of Computer Science and Systems Engineering, University of Zaragoza, Zaragoza, Spain; ^4^Interactive Systems, Adaptivity, Autonomy and Cognition Lab, Aragón Institute of Engineering Research, University of Zaragoza, Zaragoza, Spain

**Keywords:** Turing test, interaction, sensorimotor contingencies, reciprocity, perceptual crossing

In the published article, there was an error in Figure 1 as published. The probability of success for block 3 in the bot condition should be 0.70, as correctly stated in Table 1 and the second paragraph of the **Results** section. However, in Figure 1, this value is incorrectly shown as 0.95. The corrected Figure 1 and its caption appear below.

**Figure 1:**
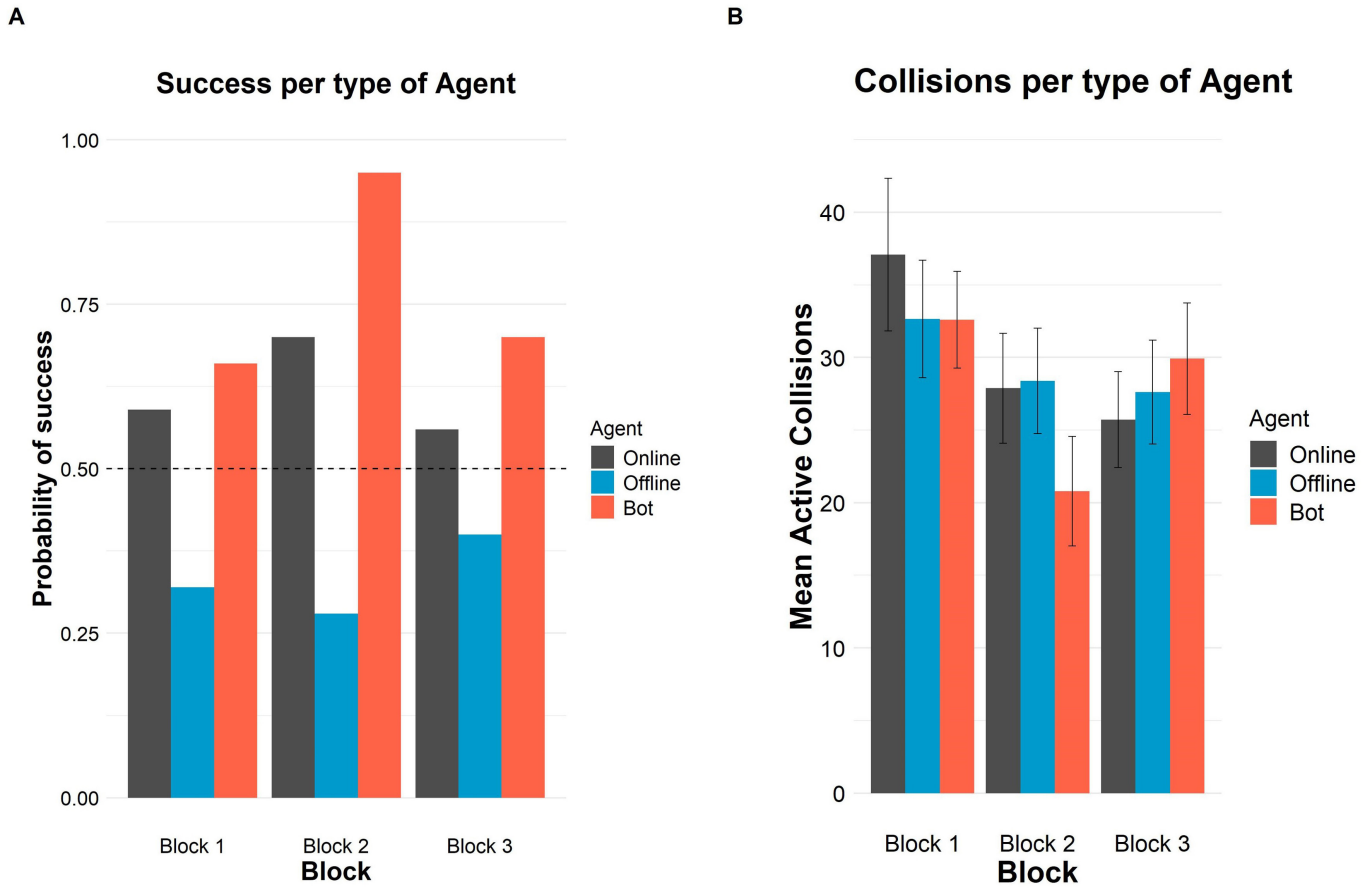
Successes and crossings per type of agent and block in Study 1. **(A)** Probability of success in each block per type of agent. The horizontal dashed line represents chance level (50%). **(B)** Mean number of crossings in each block per type of agent. Error bars depict 95% confidence intervals.

The authors apologize for this error and state that this does not change the scientific conclusions of the article in any way. The original article has been updated.

