# Supplementary material for: A Minimal Turing Test: Reciprocal Sensorimotor Contingencies for Interaction Detection
Source: Front Hum Neurosci. 2020 Mar 24;14:102. doi: 10.3389/fnhum.2020.00102 (PMC7105611; doi:10.3389/fnhum.2020.00102)
Supplement: Supplementary file 1 [file Data_Sheet_1.docx]

Supplementary Material

# Experimental procedure

The experimental platform paralleled the one designed by Bedia et al. (2014; see the supplementary material of this paper).The experimental set up is depicted in figure 1. Briefly, the participant wore headphones and used his/her computer mouse to move its avatar along a horizontal line. The other avatar on the line is moved by another participant (human online) or by the computer. The computer could implement two behaviors: an oscillatory bot or a human offline.


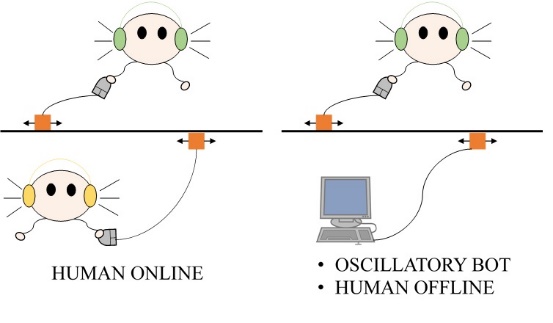


Supplementary Figure 1. Schematic illustration of the experimental set-up.

## Coding and analysis

For fractal analysis, we used a detrended fluctuation analysis (DFA) algorithm which is a method for determining the statistical self-affinity of a signal (Peng et al., 2000). In a nutshell, the DFA algorithm takes a time-series of data and divides it into boxes of equal length *n*. For each box and each value of *n*, the least squares line (the trend of the signal within the box) that best fits the data is extracted. Next, the characteristic size of the fluctuation *F(n)* is computed as the root mean square deviation between the integrated signal and its trend in each box. This computation is repeated for boxes of different size of *n*. When finished, it can be verified that *F(n)* increases with *n*. In the case of this relationship between *F(n)* vs *n* is a linear relationship on a log-log plot, then we can say that self-affinity exists in the signal.

The DFA algorithm (i) is able to determine in which range of scales the self-affinity is presented in the analyzed signal; and (ii) the value of the slope α of this relationship is a generalization of the Hurst exponent that allows us to know how self-affinity is in long-term positive autocorrelation processes. Using this parameter, it is possible to characterize if there is no correlation of a variable with its past value or if the high positive or negative movements in the present will be followed by high positive and negative movement in the future, respectively.

Since we are interested in knowing whether the experimental series are long memory dependence processes, the slope of the line which best fits the plot of log *F(scale)* versus log scale shows us the type of processes. Therefore, the DFA algorithm fits perfectly to what we are interested in testing in our experimental analysis. As it is said in the "Experimental procedure" section, the time-length of each round was 30 seconds. The analyses were done with windows ranging from 10^-2^ to 10^1^.

Other measures explained on the main article was the window of crossings. It referred to the number of crossings counting after one crossing is produced up to a certain time after this crossing. To exemplify this measure, see table 1. Supposing a series of crossings produced at specific times (first column), we calculate the density of crossing according to different ranges of time windows (the second and third columns).

**Supplementary Table 1.** Calculation of window of crossings

| **Time of crossings** | **Density of crossings with window=1000 ms** | **Density of crossings with window=500 ms.** |
| --- | --- | --- |
| **1000 ms** | 0 (there is no crossing in the following 1000 ms) | 0 (there is no crossing in the following 500 ms) |
| **3000 ms** | 3 (crossings at 3600, 3800 and 3900 ms) | 0 |
| **3600 ms** | 2 (crossings at 3800 and 3900 ms) | 2 (crossings at 3800 and 3900 ms) |
| **3800 ms** | 1 (at 3900 ms) | 1 (at 3900 ms) |
| **3900 ms** | 0 | 0 |
| **7000 ms** | 1 (at 7300 ms) | 1 (at 7300 ms) |
| **7300 ms** | 0 | 0 |
| **9000 ms** | 0 | 0 |

# Results of Study 1

In this section, we provide additional information of the results obtained in the first study.

## Fractal indices

Table 2 summarizes all the values of *β* obtained per agent and block. In general, the mean and the standard deviation of *β* showed little variation in all categories. Tailedness and kurtosis presented more variations. In block 2, the mean showed a wide variation depending on the type of agent.

**Supplementary Table 2.** Values of *β* for each agent in each block in Study 1.

|  | BLOCK 1 | BLOCK 2 | BLOCK 3 |
| --- | --- | --- | --- |
| *β* (average) |  |  |  |
| Online | 0.17 | 0.16 | 0.25 |
| Offline | 0.18 | 0.20 | 0.21 |
| Oscillatory bot | 0.28 | 0.31 | 0.27 |
| *β* (standard deviation) |  |  |  |
| Online | 0.36 | 0.40 | 0.39 |
| Offline | 0.39 | 0.38 | 0.35 |
| Oscillatory bot | 0.43 | 0.40 | 0.46 |
| *β* (tailedness) |  |  |  |
| Online | 0.10 | 0.64 | 0.28 |
| Offline | 0.25 | 0.18 | 0.04 |
| Oscillatory bot | 0.54 | 0.04 | 0.19 |
| *β* (kurtosis) |  |  |  |
| Online | 2.91 | 3.19 | 2.80 |
| Offline | 3.44 | 3.40 | 2.74 |
| Oscillatory bot | 4.22 | 3.38 | 2.43 |

## Similarity between two series

Table 3 reports all the correlation indices as a function of the window span considered.

**Supplementary Table 3.** Correlation indices as a function of the window span in Study 1.

|  | BLOCK 1 | BLOCK 2 | BLOCK 3 |
| --- | --- | --- | --- |
| Correlation at window 50 ms |  |  |  |
| Online | 0.955 | 0.959 | 0.962 |
| Offline | 0.954 | 0.953 | 0.946 |
| Oscillatory bot | 0.966 | 0.96 | 0.966 |
| Correlation at window 125 ms |  |  |  |
| Online | 0.915 | 0.930 | 0.929 |
| Offline | 0.921 | 0.916 | 0.912 |
| Oscillatory bot | 0.933 | 0.954 | 0.935 |
| Correlation at window 250 ms |  |  |  |
| Online | 0.878 | 0.903 | 0.901 |
| Offline | 0.887 | 0.876 | 0.876 |
| Oscillatory bot | 0.907 | 0.938 | 0.910 |
| Correlation at window 500 ms |  |  |  |
| Online | 0.835 | 0.863 | 0.863 |
| Offline | 0.841 | 0.843 | 0.823 |
| Oscillatory bot | 0.870 | 0.915 | 0.874 |
| Correlation at window 1000 ms |  |  |  |
| Online | 0.781 | 0.832 | 0.820 |
| Offline | 0.795 | 0.812 | 0.780 |
| Oscillatory bot | 0.844 | 0.888 | 0.845 |
| Correlation at window 1250 ms |  |  |  |
| Online | 0.761 | 0.821 | 0.807 |
| Offline | 0.783 | 0.800 | 0.766 |
| Oscillatory bot | 0.833 | 0.878 | 0.836 |
| Correlation at window 1500 ms |  |  |  |
| Online | 0.745 | 0.811 | 0.795 |
| Offline | 0.773 | 0.789 | 0.758 |
| Oscillatory bot | 0.821 | 0.870 | 0.827 |
| Correlation at window 1750 ms |  |  |  |
| Online | 0.732 | 0.805 | 0.783 |
| Offline | 0.763 | 0.778 | 0.749 |
| Oscillatory bot | 0.808 | 0.864 | 0.819 |
| Correlation at window 2000 ms |  |  |  |
| Online | 0.720 | 0.798 | 0.774 |
| Offline | 0.752 | 0.769 | 0.742 |
| Oscillatory bot | 0.797 | 0.857 | 0.810 |
| Correlation at window 2250 ms |  |  |  |
| Online | 0.710 | 0.792 | 0.766 |
| Offline | 0.743 | 0.762 | 0.734 |
| Oscillatory bot | 0.786 | 0.851 | 0.802 |
| Correlation at window 2500 ms |  |  |  |
| Online | 0.701 | 0.786 | 0.757 |
| Offline | 0.734 | 0.756 | 0.726 |
| Oscillatory bot | 0.776 | 0.844 | 0.795 |

## Statistical analysis

Table 4 provides the values resulting from the analysis of all rounds. The predictors included in our model are participants’ age and gender, *β*, density of crossings at two time points and correlation between series with windows at 500 and 1200 milliseconds. Many of the predictors (i.e., age, gender, *β* and density of crossings) did not discriminate when the participant gave the correct answer (all *p* values > 0.05). Correlation indices between series with windows at 500 and 1200 milliseconds seem to discriminate participants’ correct answer (Corr500, t(1815) = -2.14, *p* = .03; Corr1200, t(1815) = 1.8, *p* = .06). However, the p-value for the correlations for the second window span is not significant.

**Supplementary Table 4.**

|  | Value | Std. Error | DF | t-value | p-value |
| --- | --- | --- | --- | --- | --- |
| (Intercept) | 0.6817823 | 0.06836736 | 1815 | 9.972337 | 0 |
| Age | -0.0024108 | 0.00252022 | 67 | -0.95659 | 0.3422 |
| Gender M | -0.0113301 | 0.02836148 | 67 | -0.39949 | 0.6908 |
| Beta | -0.0059591 | 0.03072491 | 1815 | -0.19395 | 0.8462 |
| Coll 100 | 0.0017288 | 0.00146558 | 1815 | 1.179631 | 0.2383 |
| Coll 1200 | -0.0011165 | 0.00076868 | 1815 | -1.45248 | 0.1465 |
| Corr 500 | -0.2078342 | 0.09701811 | 1815 | -2.14222 | 0.0323 |
| Corr 2000 | 0.1804016 | 0.09697033 | 1815 | 1.860379 | 0.063 |

Secondly, we analyzed the results according to the type of agent and block. Results, however, showed that any variable predicted the correct response (all *p* values were higher than 0.05). Tables 5 to 8 present the results for Online and Offline agents in Blocks 1 and 3.

**Supplementary Table 5.** Results for Online agent in block 1.

|  | Value | Std. Error | DF | t-value | p-value |
| --- | --- | --- | --- | --- | --- |
| (Intercept) | 0.6485570 | 0.21910024 | 135 | 2.9600928 | 0.0036 |
| Age | -0.0035147 | 0.00759301 | 67 | -0.4628867 | 0.6449 |
| Gender M | -0.0456720 | 0.08687054 | 67 | -0.5257483 | 0.6008 |
| Beta | -0.1186138 | 0.10868048 | 135 | -1.0913991 | 0.2770 |
| Coll 100 | -0.0006372 | 0.00312045 | 135 | -0.2042116 | 0.8385 |
| Coll 1200 | -0.0010343 | 0.00203084 | 135 | -0.5093023 | 0.6114 |
| Corr 500 | 0.0277675 | 0.27771946 | 135 | 0.0999839 | 0.9205 |
| Corr 2000 | 0.1006210 | 0.25844201 | 135 | 0.3893369 | 0.6976 |

**Supplementary Table 6.** Results for Online agent in block 3.

|  | Value | Std. Error | DF | t-value | p-value |
| --- | --- | --- | --- | --- | --- |
| (Intercept) | 0.5532741 | 0.20935388 | 135 | 2.6427695 | 0.0092 |
| Age | -0.0020271 | 0.00774398 | 67 | -0.2617593 | 0.7943 |
| Gender M | 0.0569759 | 0.08677110 | 67 | 0.6566230 | 0.5137 |
| Beta | 0.0650999 | 0.09489566 | 135 | 0.6860156 | 0.4939 |
| Coll 100 | 0.0015385 | 0.00682399 | 135 | 0.2254496 | 0.8220 |
| Coll 1200 | 0.0004163 | 0.00328023 | 135 | 0.1268980 | 0.8992 |
| Corr 500 | -0.1004320 | 0.27055484 | 135 | -0.3712076 | 0.7111 |
| Corr 2000 | 0.1234701 | 0.28236515 | 135 | 0.4372710 | 0.6626 |

**Supplementary table 7.** Results for Offline agent in block 1.

|  | Value | Std. Error | DF | t-value | p-value |
| --- | --- | --- | --- | --- | --- |
| (Intercept) | 0.3673192 | 0.21701772 | 135 | 1.6925770 | 0.0928 |
| Age | -0.0008555 | 0.00722927 | 67 | -0.1183398 | 0.9062 |
| Gender M | -0.0192497 | 0.08257183 | 67 | -0.2331265 | 0.8164 |
| Beta | 0.1228617 | 0.09185324 | 135 | 1.3375868 | 0.1833 |
| Coll 100 | -0.0004041 | 0.00489123 | 135 | -0.0826094 | 0.9343 |
| Coll 1200 | 0.0001785 | 0.00213284 | 135 | 0.0837025 | 0.9334 |
| Corr 500 | -0.2018611 | 0.31458026 | 135 | -0.6416839 | 0.5222 |
| Corr 2000 | 0.1623919 | 0.28348747 | 135 | 0.5728362 | 0.5677 |

**Supplementary Table 8.** Results for Offline agent in block 3.

|  | Value | Std. Error | DF | t-value | p-value |
| --- | --- | --- | --- | --- | --- |
| (Intercept) | 0.4682796 | 0.20313451 | 135 | 2.3052686 | 0.0227 |
| Age | -0.0015027 | 0.00802852 | 67 | -0.1871662 | 0.8521 |
| Gender M | -0.1259986 | 0.09158138 | 67 | -1.3758102 | 0.1735 |
| Beta | -0.0704348 | 0.10494074 | 135 | -0.6711866 | 0.5032 |
| Coll 100 | 0.0067148 | 0.00462472 | 135 | 1.4519327 | 0.1488 |
| Coll 1200 | -0.0040158 | 0.00249495 | 135 | -1.6095546 | 0.1098 |
| Corr 500 | 0.2337638 | 0.24447658 | 135 | 0.9561806 | 0.3407 |
| Corr 2000 | -0.1921976 | 0.24658321 | 135 | -0.7794433 | 0.4371 |

# Results of Study 2

This section contains additional information of the results obtained in the second study.

## Participants’ responses and number of crossings

A linear mixed effect showed that the type of block had a significant effect on crossings, χ2(7) = 21.75, *p* < .001, but not the type of agent, χ2(8) = 3.06, *p* = .08 (see supplementary figure 2). Most important, the block × agent interaction was not significant, χ2(10) = 4.79, *p* = .09. The two contrasts for block showed a significant effect on crossings when comparing auditory blocks (blocks 1 and 3) with audiovisual block *b* = 3.58, *t*(94) = 4.29, *p* < .0001; and a significant effect when comparing block 1 vs block 3 *b* = 3.54, *t*(94) = 2.45, *p* = .02. The contrast for agent did not show a significant effect on crossings, *b* = 1.41, *t*(141) = 1.78, *p* = .08.


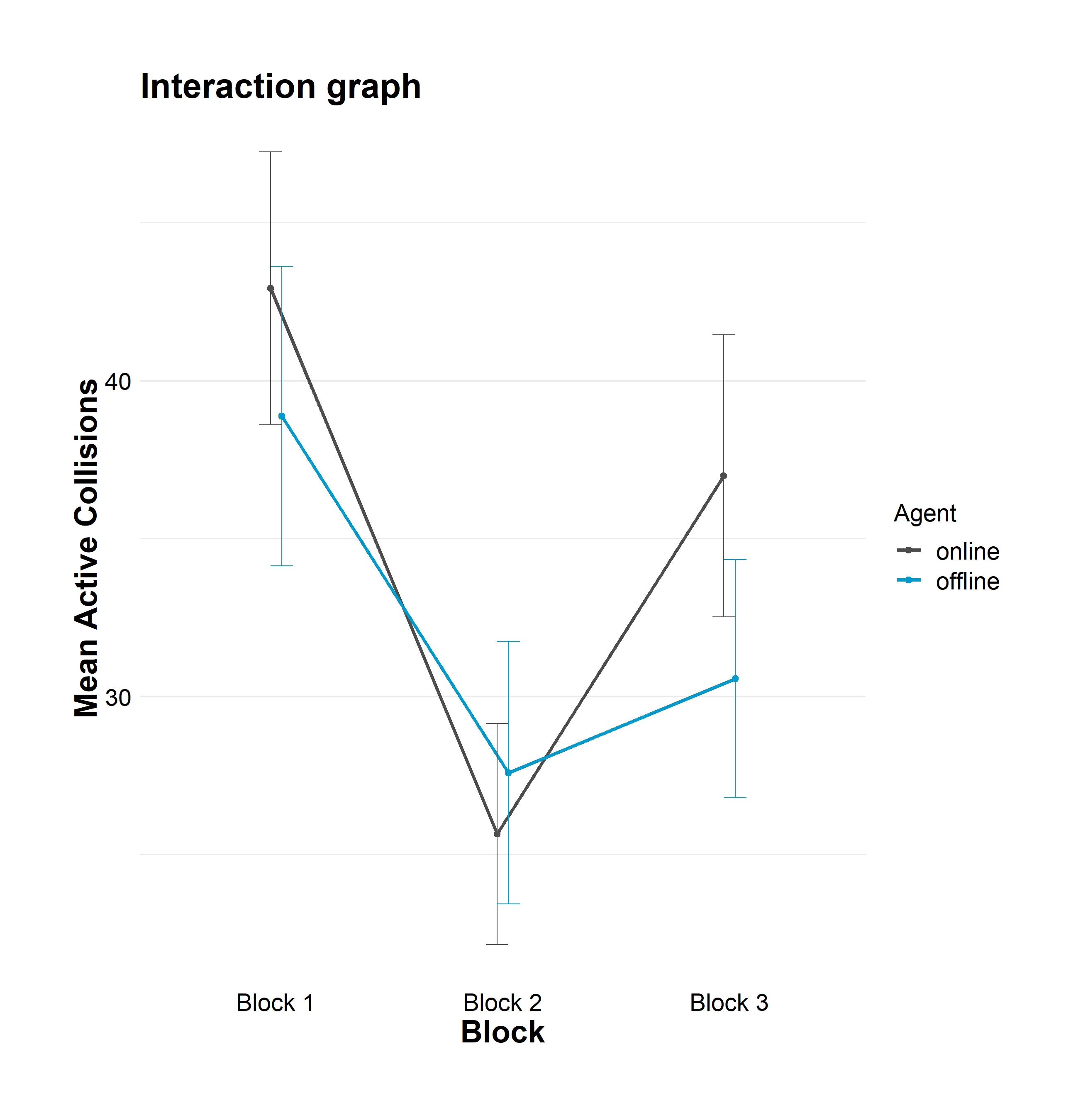


Supplementary Figure 2. Plot of the interaction between block and agent in Study 2.

## Fractal indices

Table 9 presents all the values of *β* per agent in each block. In general, the mean and the standard deviation of *β* showed little variation in all categories. Tailedness and kurtosis presented more variations.

**Supplementary Table 9.** Values of *β* for each agent in each block in Study 2

|  | BLOCK 1 | BLOCK 2 | BLOCK 3 |
| --- | --- | --- | --- |
| *β* (average) |  |  |  |
| Online | 0.28 | 0.26 | 0.23 |
| Offline | 0.34 | 0.23 | 0.20 |
| *β* (standard deviation) |  |  |  |
| Online | 0.53 | 0.38 | 0.47 |
| Offline | 0.51 | 0.41 | 0.51 |
| *β* (tailedness) |  |  |  |
| Online | 0.41 | -0.12 | 0.32 |
| Offline | 0.12 | 0.38 | 0.14 |
| *β* (kurtosis) |  |  |  |
| Online | 2.30 | 2.44 | 2.90 |
| Offline | 2.36 | 2.60 | 2.43 |

## Similarity between two series

In Table 10 we can see all correlation indices as a function of the window span considered.

**Supplementary Table 10.** Correlation indices as a function of the window span in Study 2.

|  | BLOCK 1 | BLOCK 2 | BLOCK 3 |
| --- | --- | --- | --- |
| Correlation at window 50 ms |  |  |  |
| Online | 0.962 | 0.969 | 0.959 |
| Offline | 0.963 | 0.965 | 0.957 |
| Correlation at window 125 ms |  |  |  |
| Online | 0.928 | 0.946 | 0.925 |
| Offline | 0.934 | 0.947 | 0.921 |
| Correlation at window 250 ms |  |  |  |
| Online | 0.901 | 0.924 | 0.894 |
| Offline | 0.907 | 0.923 | 0.890 |
| Correlation at window 500 ms |  |  |  |
| Online | 0.867 | 0.891 | 0.856 |
| Offline | 0.873 | 0.883 | 0.849 |
| Correlation at window 1000 ms |  |  |  |
| Online | 0.821 | 0.848 | 0.811 |
| Offline | 0.830 | 0.846 | 0.809 |
| Correlation at window 1250 ms |  |  |  |
| Online | 0.807 | 0.836 | 0.793 |
| Offline | 0.817 | 0.831 | 0.809 |
| Correlation at window 1500 ms |  |  |  |
| Online | 0.796 | 0.828 | 0.781 |
| Offline | 0.806 | 0.820 | 0.785 |
| Correlation at window 1750 ms |  |  |  |
| Online | 0.784 | 0.820 | 0.771 |
| Offline | 0.797 | 0.810 | 0.775 |
| Correlation at window 2000 ms |  |  |  |
| Online | 0.774 | 0.813 | 0.762 |
| Offline | 0.788 | 0.798 | 0.767 |
| Correlation at window 2250 ms |  |  |  |
| Online | 0.763 | 0.804 | 0.753 |
| Offline | 0.779 | 0.792 | 0.760 |
| Correlation at window 2500 ms |  |  |  |
| Online | 0.754 | 0.798 | 0.746 |
| Offline | 0.769 | 0.748 | 0.756 |

## Statistical analysis

Table 11 provides the values resulting from the analysis of all rounds. The predictors included in our model are type of agent, type of block, participants’ age and gender, *β*, density of crossings at two time points and correlation between series with windows at 500 and 1200 milliseconds. On the one hand, block 2 discriminated participants’ correct answer (t(837) = 3.53, *p* < .001). On the other hand, all the other predictors (type of agent, age, gender, beta, density of crossings and correlation indices) did not discriminate when the participant gave the correct answer (*p* > .05)

**Supplementary Table 11.**

|  | Value | Std.Error | DF | t-value | p-value |
| --- | --- | --- | --- | --- | --- |
| (Intercept) | 0.4543951 | 0.14936323 | 837 | 3.042216 | 0.0024 |
| Agent Online | -0.0083023 | 0.03240207 | 837 | -0.256227 | 0.7978 |
| Block 2 | 0.1437236 | 0.04068513 | 837 | 3.532584 | 0.0004 |
| Block 3 | -0.0024259 | 0.04022254 | 837 | -0.060313 | 0.9519 |
| Age | 0.0038288 | 0.00485194 | 837 | 0.789135 | 0.4303 |
| Gender M | 0.0216570 | 0.05678870 | 44 | 0.381362 | 0.7048 |
| Beta | 0.0106178 | 0.04164191 | 837 | 0.254979 | 0.7988 |
| Coll 100 | -0.0050630 | 0.00289876 | 837 | -1.746623 | 0.0811 |
| Coll 1200 | 0.0029073 | 0.00142061 | 837 | 2.046492 | 0.041 |
| Corr 500 | -0.1659183 | 0.17069911 | 837 | -0.971993 | 0.3313 |
| Corr 2000 | 0.1022158 | 0.15336566 | 837 | 0.666484 | 0.5053 |
